# Impact of cardiac magnetic resonance on the diagnosis of hypertrophic cardiomyopathy - a 10-year experience with over 1000 patients

**DOI:** 10.1007/s00330-020-07207-8

**Published:** 2020-09-02

**Authors:** Mateusz Śpiewak, Mariusz Kłopotowski, Natalia Ojrzyńska, Joanna Petryka-Mazurkiewicz, Barbara Miłosz-Wieczorek, Łukasz Mazurkiewicz, Jacek Grzybowski, Zofia Bilińska, Adam Witkowski, Magdalena Marczak

**Affiliations:** 1 Magnetic Resonance Unit, National Institute of Cardiology, ul. Alpejska 42, 04-628 Warsaw, Poland; 2 Department of Cardiology and Interventional Angiology, National Institute of Cardiology, Warsaw, Poland; 3 Department of Cardiomyopathies, National Institute of Cardiology, Warsaw, Poland; 4 Department of Coronary Artery Disease and Structural Heart Diseases, National Institute of Cardiology, Warsaw, Poland; 5 Unit for Screening Studies in Inherited Cardiovascular Diseases, National Institute of Cardiology, Warsaw, Poland

**Keywords:** Hypertrophic cardiomyopathy, Magnetic resonance imaging, Hypertension, Ultrasonography, Echocardiography

## Abstract

**Objectives:**

To assess the value of cardiac MRI in comparison to echocardiography in consecutive patients with previously diagnosed and new suspected hypertrophic cardiomyopathy (HCM).

**Methods:**

All MRI studies of patients with HCM or suspected disease performed at our centre within a 10-year time period were evaluated. Initial diagnoses (echocardiography-based) and final (MRI-based) diagnoses were compared in subgroups, and the discrepancies were recorded.

**Results:**

A total of 1006 subjects with HCM or suspected HCM were identified (61% males, 39% females; median age, 49.1 years; interquartile range, 34.9–60.4). In 12 (2.2%) out of 550 patients with known HCM, MRI indicated a diagnosis other than HCM, including but not limited to the subaortic membrane (*n* = 1, 8.3%) or mild left ventricular hypertrophy (*n* = 5, 41.7%). Among all patients with suspected HCM (*n* = 456), MRI diagnosis was different from HCM in 5.3% (*n* = 24) of patients. In an additional 20.4% of patients (*n* = 93), no significant hypertrophy was present. In total, among patients with suspected HCM, MRI led to clear HCM diagnosis in 204 (44.7%) patients. Among patients with a history of uncontrolled hypertension suspected of having HCM, MRI aided in identifying cardiomyopathy in 47.9% of patients. This subgroup contained the largest proportion of patients with an ambiguous diagnosis, namely, 29.6% compared with 13.8% in the remaining groups of patients with suspected HCM (*p* = 0.0001).

**Conclusions:**

In a small but important group of patients with ultrasound-based HCM, cardiac MRI can diagnose previously unknown conditions and/or refute suspected cardiomyopathy. The diagnostic yield of MRI when compared to echocardiography in patients suspected of having HCM is 44.7%.

**Key Points:**

*• Out of 550 patients previously diagnosed with echocardiography but without magnetic resonance imaging (MRI) as having hypertrophic cardiomyopathy (HCM), we diagnosed a different disease in 12 (2.2%) patients using MRI.*

*• Among patients with suspected HCM based on echocardiography, MRI led to clear HCM diagnosis in 44.7% of patients.*

*• In patients with a history of uncontrolled hypertension suspected, based on an echocardiogram, of having HCM, MRI aided in identifying cardiomyopathy in 47.9% of patients. This subgroup contained the largest proportion of patients with an ambiguous diagnosis.*

## Introduction

Cardiac magnetic resonance imaging (MRI) has revolutionised our understanding and management of various cardiovascular diseases. Because of its excellent ability to non-invasively characterise tissue, cardiac MRI has emerged as particularly useful in patients with cardiomyopathies, including patients with suspected or confirmed hypertrophic cardiomyopathy (HCM) [1–3]. MRI is useful not only in patients with poor acoustic windows and inadequate image quality of an ultrasound study but also for the differential diagnosis of left ventricular hypertrophy (LVH), which can be caused by several conditions including HCM, Fabry disease or cardiac amyloidosis [3]. If HCM is unrecognised or misdiagnosed, HCM patients remain at risk for sudden cardiac death (SCD), and family screening cannot be implemented. Thus, confirming HCM diagnosis or discerning sarcomeric HCM from infiltrative/metabolic disorders at the early stages of diagnostic workup is critical. Accordingly, we aimed to assess the value of cardiac MRI when compared to echocardiography in daily practice in a large contemporary cohort of consecutive patients with HCM or suspected of having this cardiomyopathy. We hypothesised that cardiac MRI would be able to uncover previously unknown disease in patients with HCM established by echocardiography. Additionally, we hypothesised that cardiac MRI would allow a definitive diagnosis of HCM to be made in a substantial group of patients suspected to have HCM.

## Methods

### Study population

The MRI unit was established at our institution in March 2008, and the time period included in the analysis spanned 10 years (March 2008–March 2018). All consecutive patients referred for MRI studies with an echocardiography-based diagnosis of HCM or suspected of having HCM based on echocardiogram were included. All MRI studies were reviewed by a single operator with considerable experience in assessing MRI in HCM patients (cardiac MRI level 3 certified expert). In doubtful cases, MRI diagnosis was performed by consensus among three cardiologists (all of whom had experience and level 3 training in cardiac MRI) and a radiologist. Referral forms for MRI studies were inspected, and patients were categorised into the following groups based on the information given in the referral and transthoracic echocardiography (initial—pre-MRI—diagnosis): (a) established (known) HCM or (b) suspected HCM. The latter category included the following subcategories: patients with a family history of HCM, referrals concerning differential diagnosis of HCM vs LVH secondary to uncontrolled hypertension or HCM vs cardiac amyloidosis or storage disease and patients in whom cardiac tumour mimicking HCM was suspected.

Final diagnoses made based on MRI studies were categorised as follows: (1) definitive diagnosis of HCM (confirmation of HCM), (2) definitive diagnosis other than HCM causing LVH (refuted diagnosis of HCM, e.g. LVH due to other causes such as hypertension, athlete’s heart, aortic stenosis), (3) previously unknown infiltrative/storage disease (e.g. cardiac amyloidosis, Fabry disease), (4) cardiac tumour, (5) equivocal diagnosis (borderline LVH, overlapping phenotypes, no LVH present but other abnormalities such as myocardial crypts or papillary muscle abnormalities present) or (6) normal MRI scan.

### Ethics approval

Patients who were included prospectively (recruited from 2015) provided written informed consent, and approval was granted by the local ethics committee for the retrospective analyses (Reference number: 1656).

### Criteria for MRI diagnoses

A definitive diagnosis of HCM was made when the pattern of LVH was clearly asymmetrical with a maximal LV wall thickness ≥ 15 mm in the absence of other causes of LV hypertrophy of that magnitude [1, 2]. In subjects with a family history of HCM, a lesser degree of LVH was diagnostic (≥ 13 mm). Particularly, a definitive diagnosis of HCM was made in patients demonstrating non-contiguous areas of hypertrophy [4]. Additionally, the presence and patterns of LGE were analysed and aided in the differentiation of HCM from HCM mimics. Although there is no pathognomonic LGE pattern for HCM, patchy mid-wall LGE at the anterior and posterior right ventricular (RV) insertion points and in segments with maximum LV thickening assisted in the diagnosis of HCM [1, 5, 6].

In patients with a history of hypertension, symmetrical LVH not fulfilling the diagnostic criteria for HCM in terms of the magnitude of wall thickness favoured the diagnosis of LVH secondary to hypertension [1, 5]. When the maximal LV wall thickness in this subgroup was greater than or equal to 15 mm, the presence of the additional morphological abnormalities and the LGE pattern were employed in the differentiation of hypertensive heart disease and HCM [5, 7–9]. If ambiguous findings precluded a clear distinction of these two conditions, then the MRI-derived diagnosis was classified as equivocal.

In cases of symmetric LVH, particular care was taken to identify hallmark features suggestive of storage/infiltrative disease such as RV hypertrophy and typical patterns of LGE (mid-wall inferolateral LGE suggestive of Fabry disease; suboptimal myocardial nulling, global subendocardial or transmural LGE distribution with a non-coronary pattern—all virtually pathognomonic for cardiac amyloidosis) [5, 10]. The presence of a thickened intra-atrial septum and pericardial effusion were further indicators of a diagnosis of cardiac amyloidosis.

The diagnosis of cardiac tumour was made based on typical pre- and postcontrast tumour morphology and signal characteristics [11].

### Image acquisition and analysis

MRI studies were performed on a 1.5-T scanner (Avanto/Avanto^fit^, Siemens). All MRI studies included assessments of myocardial function using cine sequences. To reveal normal clinical scenarios, none of the MRI studies were excluded due to quality reasons. In all patients without contraindications, a commercially available gadolinium-based contrast agent was administered intravenously at a standard dosage (0.1 mmol/kg), and the presence of LGE was adjudicated based on one of the standard imaging sequences in long-axis and short-axis orientations covering the entire myocardium. LV end-diastolic and end-systolic volumes (LVEDV and LVESV, respectively), mass (LVM) and ejection fraction (LVEF) were calculated from a stack of short-axis images covering the ventricles from the base to the apex. Volume and mass calculations were indexed for body surface area. Maximal LV wall thickness was determined in each patient in short-axis slices.

### Statistical analysis

Continuous data were checked for a normal distribution using the Kolmogorov–Smirnov test and are presented as medians with the interquartile range (for data without a normal distribution). Comparisons between more than two subgroups were performed using the Kruskal–Wallis test. Categorical variables are presented as numbers and percentages and were compared using the chi-squared test. A two-tailed *p* value of less than 0.05 was considered to indicate statistical significance. All statistical analyses were performed with MedCalc 19.1.3 software (MedCalc Software Ltd).

## Results

### Baseline characteristics

Out of 8630 cardiac MRI studies, we selected 1422 studies that included patients who were referred due to HCM, suspected HCM, LVH of unknown aetiology, cardiac amyloidosis or storage disease. Upon excluding repeated studies and studies of patients who were referred due to clinical presentation highly suggestive of cardiac amyloidosis or Fabry disease, we formed a group of 1060 patients. Subsequently, we excluded patients with a history of any type of septal reduction therapy (*n* = 21) or who were younger than 18 years (*n* = 33), leaving 1006 individuals for the final analysis [614 (61%) males, 392 (39%) females; median age, 49.1 years; interquartile range, 34.9–60.4 years; range, 18.0–84.8 years]. The baseline characteristics of the study group divided according to initial (pre-MRI, based on echocardiography) diagnoses are presented in Table 1 and according to the final diagnoses in Table 2.

**Table 1:** Baseline characteristics of patients divided according to initial diagnosis (pre-MRI) (*n* = 1006)

	Patients with known HCM (*n* = 550)	Patients with suspected HCM (*n* = 456)					
		Family history of HCM (*n* = 108)	Differential diagnosis between HCM and HHD (*n* = 71)	Differential diagnosis between HCM and storage/infiltrative disease (*n* = 71)	Differential diagnosis between HCM and cardiac tumour (*n* = 4)	Other cases of suspected HCM/LVH of unknown aetiology (*n* = 202)	*p* value
Age (years)	50.5 (37.3–60.4)	33.7 (27.6–43.2)	55.4 (48.9–65.4)	50.1 (40.6–62.8)	Range 18–33	49.2 (32.3–61.6)	< 0.000001
Sex (% males) (males/females)	56% (308/242)	68% (73/35)	73% (52/19)	70% (50/21)	75% (3/1)	63% (128/74)	0.004
Maximal LV wall thickness in MRI (mm)	22 (19–25)	14 (11–16)	18 (15–21)	18 (15–23)	Range 11–40	15 (11–17)	< 0.000001
Maximal LV wall thickness in echocardiography (mm)	21 (18–25)	13 (11–15)	17 (15–21)	18 (15–22)	Range 10–40	15 (13–18)	< 0.000001
LVEDV (mL/m^2^)	87 (76–99)	83 (74–91)	88 (76–98)	85 (72–106)	Range 75–107	85 (73–97)	0.33
LVESV (mL/m^2^)	28 (22–34)	29 (24–35)	28 24–38)	36 (24–47)	Range 17–42	29 (22–39)	0.09
LVEF (%)	67 (62–73)	64 (60–67)	67 (61–71)	60 (48–69)	Range 59–80	64 (58–71)	0.000002
LGE (%)*	87% (471/539)	38% (41/107)	69% (47/68)	81% (55/68)	67% (2/3)	50% (98/196)	0.004

Unless otherwise specified, data are presented as absolute numbers and percentages or medians with the interquartile range. The Kruskal–Wallis test was performed with the exclusion of patients in whom MRI aided in the differential diagnosis between HCM and cardiac tumour

*HCM* hypertrophic cardiomyopathy, *HHD* hypertensive heart disease, *LV* left ventricular, *LVH* left ventricular hypertrophy, *LVEDV* left ventricular end-diastolic volume, *LVEF* left ventricular ejection fraction, *LVESV* left ventricular end-systolic volume, *LGE* late gadolinium enhancement, *MRI* magnetic resonance imaging

*There were 25 patients (2.5%) in whom gadolinium-based contrast agent was not administered

**Table 2:** Characteristics of patients divided according to final diagnosis (MRI-based) (*n* = 1006)

	Definitive diagnosis of HCM (*n* = 742)	Definitive diagnosis other than HCM causing LVH (*n* = 64)	Previously unknown infiltrative/storage disease (*n* = 17)	Cardiac tumour (*n* = 1)	Equivocal diagnosis (*n* = 74)	Normal MRI scan (*n* = 72)	Diagnosis different from HCM (*n* = 36)	*p* value
Age	50.4 (40.0–61.2)	43.5 (31.3–58.1)	55.4 (47.7–63.2)	25.2	47.4 (33.5–60.0)	32.8 (22.9–46.1)	48.3 (37.5–64.5)	< 0.000001
Sex (% males) (males/females)	58% (430/312)	81% (52/12)	76% (13/4)	100% (1/0)	70% (52/22)	63% (45/27)	58% (21/15)	0.001
Maximal LV wall thickness in MRI (mm)	21 (18–25)	13 (12–15)	17 (15–20)	40	15 (13–18)	10 (10–11)	13 (10–14)	< 0.000001
Maximal LV wall thickness in echocardiography (mm)	20 (17–24)	15 (13–17)	18 (16–21)	40	16 (13–18)	11 (10–14)	14 (12–18)	< 0.000001
LVEDV (mL/m^2^)	85 (75–97)	90 (77–104)	90 (74–109)	75	87 (79–97)	83 (73–96)	94 (81–114)	0.01
LVESV (mL/m^2^)	28 (22–35)	31 (25–39)	40 (30–63)	23	31 (24–37)	31 (25–39)	42 (30–69)	< 0.000001
LVEF (%)	67 (62–73)	63 (58–69)	54 (42–65)	70	65 (60–70)	61 (57–66)	55 (41–63)	< 0.000001
LGE (%)*	85% (619/728)	24% (15/62)	100% (16/16)	100% (1/0)	58% (40/69)	0	68% (23/34)	< 0.0001

Unless otherwise specified, data are presented as absolute numbers and percentages or medians with the interquartile range. The Kruskal–Wallis test was performed with the exclusion of a patient whose MRI-based diagnosis was cardiac tumour

*HCM* hypertrophic cardiomyopathy, *HHD* hypertensive heart disease, *LV* left ventricular, *LVH* left ventricular hypertrophy, *LVEDV* left ventricular end-diastolic volume, *LVEF* left ventricular ejection fraction, *LVESV* left ventricular end-systolic volume, *LGE* late gadolinium enhancement, *MRI* magnetic resonance imaging

*There were 25 patients (2.5%) in whom gadolinium-based contrast agent was not administered

### Impact of MRI on the diagnosis of patients with known HCM

**Fig. 1:**
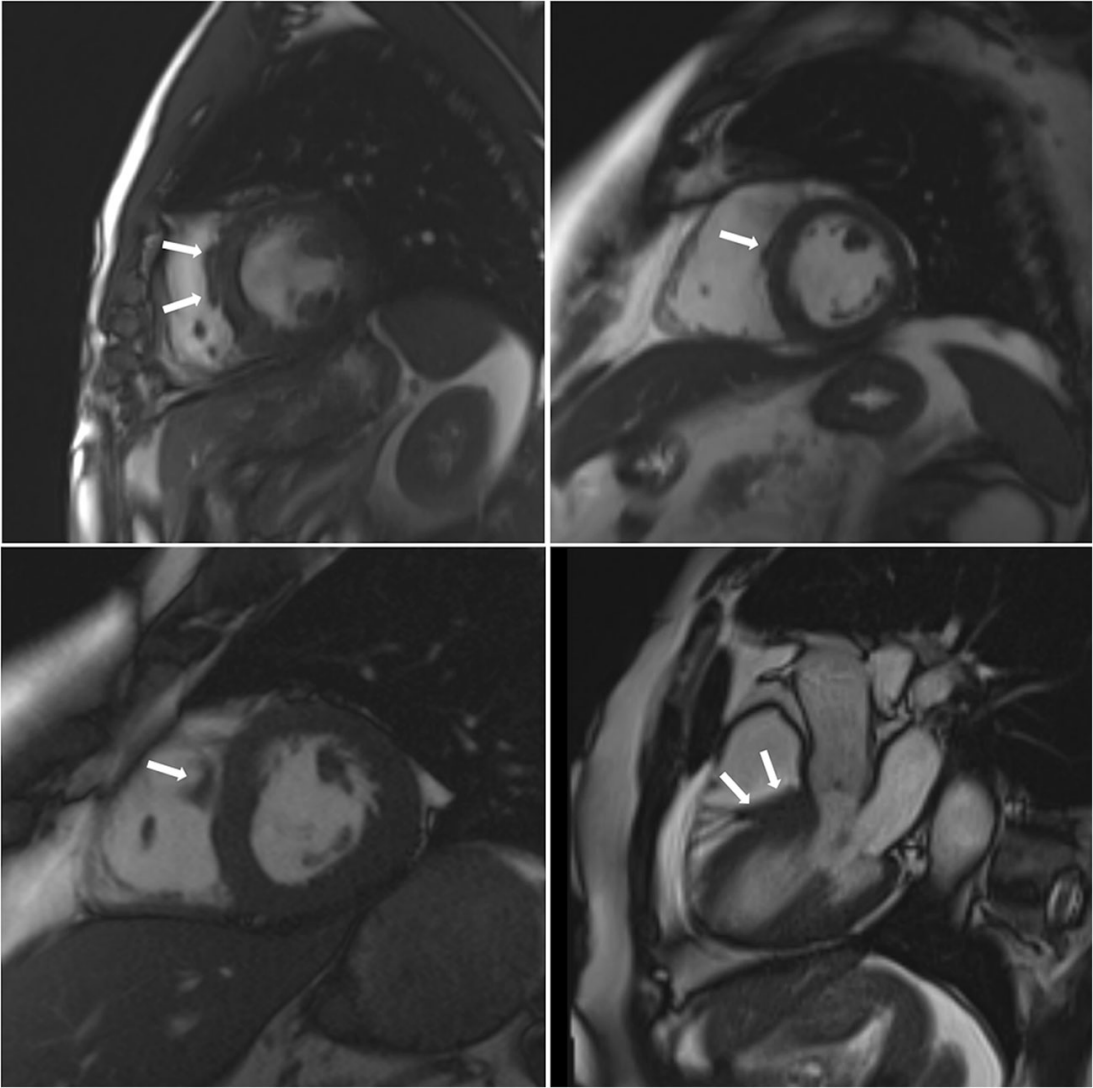
Right ventricular trabeculae (arrows) may have led to inaccurate echo measurement of left ventricular wall thickness

**Fig. 2:**
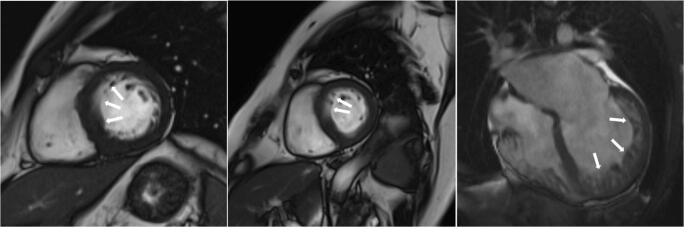
Left ventricular trabeculae (arrows) may have led to inaccurate echo measurement of left ventricular wall thickness

**Fig. 3:**
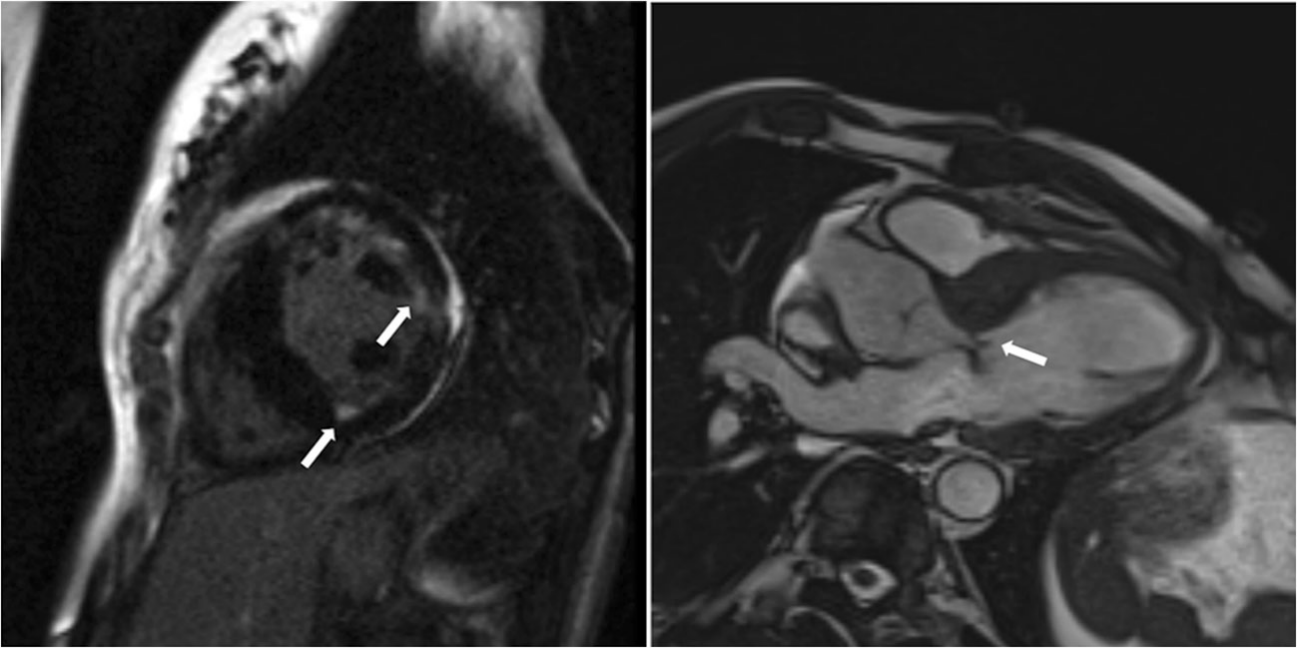
Causes of severe left ventricular hypertrophy other than hypertrophic cardiomyopathy. Left: late gadolinium enhancement present in the subendocardium of the anterior and lateral wall and at the right ventricular inferior junction point (arrows); the patterns of late gadolinium enhancement are atypical for sarcomeric hypertrophic cardiomyopathy; and storage disease was suggested (subsequently Danon disease was recognised). Right: subaortic membrane (arrow)

**Fig. 4:**
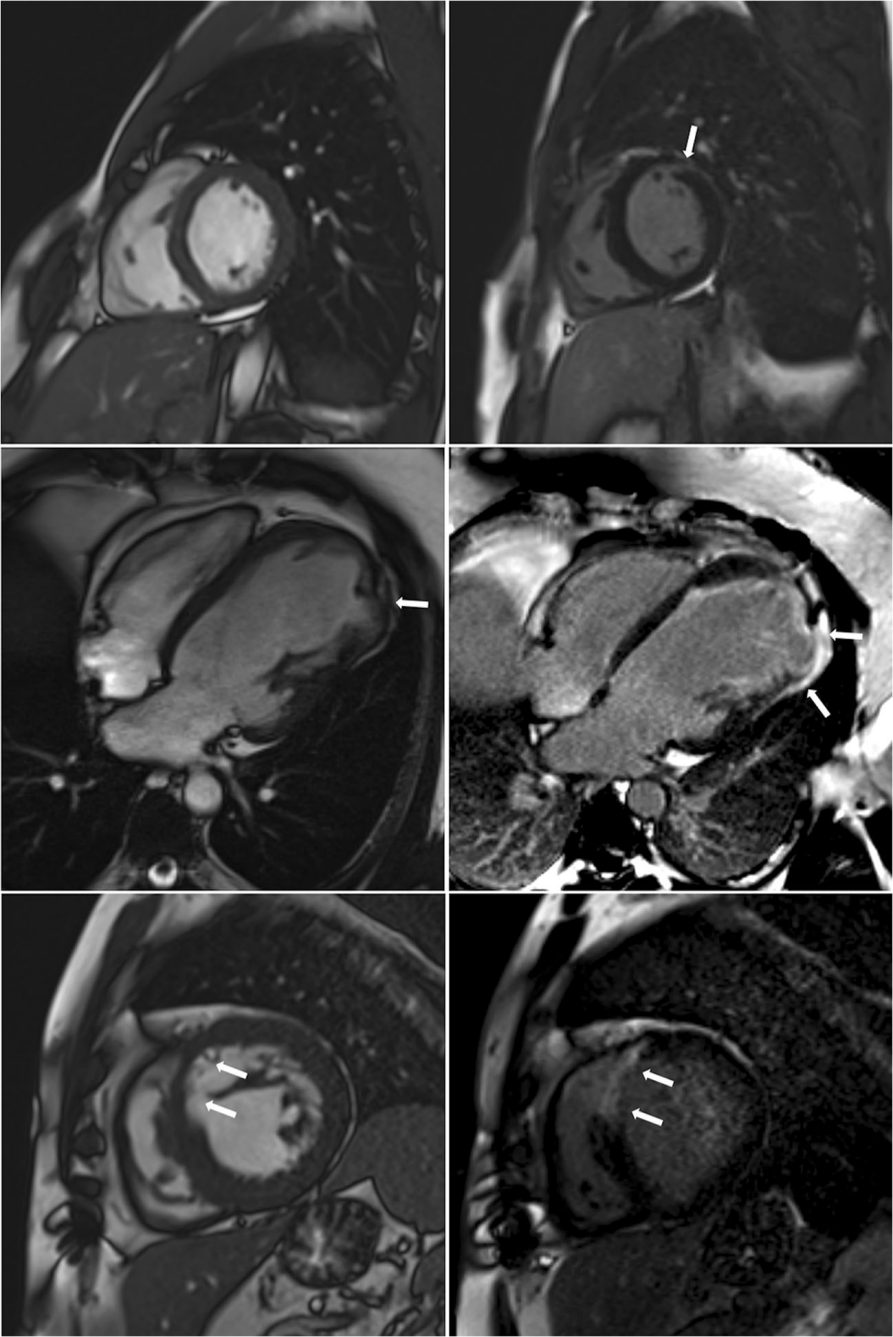
Diagnoses other than hypertrophic cardiomyopathy. Top row: left: only mild left ventricular hypertrophy with late gadolinium enhancement presenting a non-ischaemic pattern (right, arrow, status post-myocarditis). Middle row: left: aneurysm of the lateral wall apical segment (arrows) mimicking left ventricular hypertrophy in echocardiography; right: aneurysm of the lateral wall apical segment with transmural late gadolinium enhancement (arrows). Bottom row: left: “asymmetric” left ventricular hypertrophy due to prior myocardial infarction with subsequent wall thinning (arrows); right: transmural late gadolinium enhancement presenting an ischaemic pattern

There were 550 patients with known HCM referred for MRI. In 12 of these patients (2.2%), MRI indicated a diagnosis other than HCM, including trabeculae which may have led to overestimation of interventricular thickness, subaortic membrane, LV aneurysm and unrecognised myocardial infarction (Table 3, Figs. 1, 2, 3 and 4).

**Table 3:** Characteristics of patients with an established (pre-MRI, based on echocardiography) diagnosis of HCM in whom cardiac MRI revealed other diagnoses

	Sex	Age at MRI study (years)	Maximal wall thickness at echocardiography (mm)	Pre-MRI diagnosis	Maximal wall thickness at MRI (mm)	MRI diagnosis	Figure number with representative image
1	Male	27.5	16	HCM	13	Mild LVH secondary to hypertension	Fig. 1, top left
2	Male	64.2	16	HCM	12	Mild LVH secondary to hypertension	Fig. 1, top right
3	Male	44.8	16	HCM	13	Mild LVH secondary to hypertension	Fig. 1, bottom left
4	Female	67.6	18	HCM	14	LVH secondary to hypertension	Fig. 1, bottom right
5	Male	44.5	16	HCM	13	Mild LVH secondary to hypertension	Fig. 2, left
6	Female	72.0	18	HCM	12	Mild LVH secondary to hypertension	Fig. 2, middle
7	Male	21.3	18	HCM with systolic dysfunction	12	LVNC with systolic dysfunction	Fig. 2, right
8	Female	28.6	20	HCM	17	Storage disease (subsequently, Danon disease was recognised)	Fig. 3, left
9	Male	60.5	18	HCM with severe LVOTO	21	Subaortic membrane	Fig. 3, right
10	Male	23.4	15	HCM, history of pericarditis, abnormal ECG	12	Status post-myocarditis, mild LVH secondary to hypertension	Fig. 4, top row
11	Male	36.7	HCM diagnosed during childhood, 12 mm in the current echo	HCM during childhood, apical aneurysm	10	Apical aneurysm due to myocardial infarction or of congenital origin	Fig. 4, middle row
12	Male	47.1	19	HCM with systolic dysfunction	19	“Asymmetric” LVH due to prior myocardial infarction with subsequent wall thinning	Fig. 4, bottom row

*HCM* hypertrophic cardiomyopathy, *IVS* interventricular septum, *LGE* late gadolinium enhancement, *LVH* left ventricular hypertrophy, *LVNC* left ventricular non-compaction, *LVOTO* left ventricular outflow tract obstruction, *MRI* magnetic resonance imaging

### Impact of MRI on the diagnosis of patients with suspected HCM

Among all patients with suspected HCM (*n* = 456), 5.3% (*n* = 24) of the patients had an MRI-based diagnosis that was different from HCM (LVH with LV dilatation and systolic dysfunction, *n* = 8; left ventricular non-compaction, *n* = 5; Churg–Strauss syndrome and Loeffler’s endocarditis, *n* = 2; subaortic membrane/fibromuscular tissue, *n* = 2; aortic stenosis, *n* = 1; prior myocardial infarction, *n* = 1; excessive epicardial/intramyocardial adipose tissue, *n* = 1; congenital myocardial developmental anomaly of the interventricular septum, *n* = 1; triatrial heart with concomitant LVH, *n* = 1; partial anomalous pulmonary venous return with LVH due to renal disease, *n* = 1; and left ventricular aneurysm, *n* = 1). In an additional 20.4% of patients (*n* = 93), no significant LVH was present (maximal LV wall thickness ≤ 11 mm).

Among the 108 patients with a family history of suspected HCM, MRI enabled a definitive diagnosis of HCM in 56 (51.9%) patients, the results were borderline in 20 (18.5%) patients and required further testing, hypertensive heart disease was diagnosed in 5 (4.6%) patients and normal MRI scans were revealed in 26 (24.1%) patients. Additionally, in 1 individual (0.9%), Fabry disease was suggested based on MRI findings, which was further confirmed by biochemical and genetic testing.

HCM was diagnosed in 46.5% of patients who were referred for differential diagnosis of HCM vs infiltrative/metabolic disorders, while in 21.1% (15/71) of patients, MRI indicated cardiac amyloidosis/storage disease. There were 4 (5.6%) patients with normal MRI findings in this subgroup.

In patients with a history of uncontrolled hypertension suspected of having HCM, cardiac MRI aided in the identification of cardiomyopathy in 47.9% of the patients. This subgroup of patients with suspected HCM contained the largest proportion of patients with ambiguous diagnosis, namely, 29.6% compared with 13.8% in the remaining groups of patients with suspected HCM (*p* = 0.0001).

Four patients were referred for MRI for differentiation of HCM vs cardiac tumour. In one patient, a large cardiac fibroma was diagnosed. In one patient, neither LV mass nor LVH was observed (the patient had a normal MRI scan). One patient with LVH did not consent for contrast agent; thus, the accuracy of the final differentiation of cardiac tumour vs hypertrophied myocardium was limited. However, none of the native images raised the suspicion of cardiac tumours. Nevertheless, considering the lack of postcontrast images, the final diagnosis was categorised as equivocal. One patient with mass-like LVH was diagnosed with HCM.

In total, cardiac MRI enabled us to establish a definitive diagnosis of HCM in 204 out of 456 patients (44.7%) suspected of having cardiomyopathy.

### LGE imaging

In 25 patients (2.5%), LGE imaging was not performed either as a consequence of the patient’s refusal for contrast agent administration, contraindications due to severe renal failure or study termination prior to contrast agent injection. Among the remaining patients (*n* = 981, 97.5%), the presence of LGE was revealed in 718 individuals (73.2%) and the proportion of patients with LGE was the highest among patients with MRI-derived amyloidosis/storage disease (100%), followed by those with HCM diagnosis (85%), a diagnosis different from HCM (68%), equivocal diagnosis (58%) and a diagnosis other than HCM causing LVH (24%) (Table 2).

## Discussion

We proved in a large sample of consecutive patients with known or suspected HCM that MRI impacts diagnoses in this population. Over the 10 years evaluated, we observed significant contributions of MRI in confirming HCM, such as for identifying the disease in relatives of HCM patients and refuting HCM diagnosis in patients previously misdiagnosed as having HCM but as actually having a distinct disease mimicking HCM.

More than a decade ago, case reports, case series and small clinical studies demonstrated the valuable role of MRI in initially unexplained electrocardiogram abnormalities related to apical hypertrophy [12, 13] or LVH confined to the anterior/anterolateral LV free wall [14, 15]. These abnormalities were previously undetected by echocardiography, and only MRI permitted the visualisation of LV hypertrophy and the subsequent diagnosis of HCM. Since then, evidence of the incremental value of cardiac MRI over echocardiography for evaluating patients with HCM has continued to accumulate. Many of these studies focused on the detection, quantification and prognostic information of myocardial scarring [16–19]. Apart from the role of cardiac MRI in SCD prediction, however, there is a relative paucity of studies that have assessed the systematic clinical impact of MRI in the diagnosis of HCM in patients with suspected or confirmed HCM [4, 13, 15]. In a sample of 10 patients, Moon et al demonstrated that MRI enabled the diagnosis of apical HCM in all patients with suspected (based on ECG repolarisation changes) HCM but normal echo [13]. Maron and colleagues showed that in 12% of HCM patients, LVH in the anterolateral wall, the posterior septum or the apical region may be missed or underestimated by echocardiography [4]. Thus, in a small but important group of patients, only MRI is able to detect the HCM phenotype, contributing to a new diagnosis of HCM [4, 15].

Screening of HCM relatives remains challenging and is usually based on standard ECG and transthoracic echocardiography [1, 2]. We introduced cardiac magnetic resonance (CMR) in a family screening program since the opening of the cardiac MRI unit at our centre. We employed liberal criteria for MRI referral for relatives of HCM patients based on symptoms, ECG and echocardiography. The referral decision and its timing are at the discretion of the treating physician. In total, MRI led to a definite diagnosis of HCM in almost 50% of patients screened, and almost 25% of patients with a family history of HCM had a normal MRI scan. The lack of an HCM phenotype does not exclude the possibility that the patient is an asymptomatic (at the time of evaluation) mutation carrier presenting a completely normal MRI result (genotype positive–phenotype negative). Although a normal CMR scan cannot rule out such mutation carriers, a normal MRI scan reassures a low risk of SCD in the vast majority of patients [1]. Moreover, there are currently no specific recommendations for any treatment (apart from regular long-term follow-up) in asymptomatic HCM mutation carriers free from abnormalities in diagnostic tests, including imaging studies [1, 2]. On the other hand, even in the absence of LVH and normal ECG, MRI may provide some evidence, such as myocardial crypts and papillary muscle abnormalities, of an underlying genetic disorder [1, 20–22]. These anomalies may forecast the future development of full blown cardiomyopathy later in life and provide a rationale for the careful monitoring of such patients. There may be deleterious consequences of missed HCM diagnosis. Not only could treatment be omitted in a proband but also subsequent family screening may not be initiated. Thus, a diagnosis of cardiomyopathy may be overlooked, relatives with a clinical disease (phenotype positive) may be missed, and consequently, SCD risk assessment and SCD preventative measures, such as implantation of a cardioverter-defibrillator, may not be initiated when needed.

Out of 550 patients diagnosed previously (without MRI) as having HCM, we diagnosed a different disease in 12 (2.2%) patients using cardiac MRI. Of note, in 9 patients, either mild LVH or no LVH was observed on MRI (Table 3, patient nos. 1–7, 10 and 11; Figs. 1, 2, and 4, top and middle rows). This finding may be attributable to the fact that echocardiography may overestimate maximal LV wall thickness, leading to false diagnoses of LVH and HCM [8]. Echocardiography is operator dependent and the left ventricle wall thickness measurements are subject to observer error as well as possible inclusion of right ventricular cavity/wall elements and LV trabeculations. Additionally, one patient with a pre-MRI diagnosis of HCM had an MRI-derived diagnosis of LV non-compaction (patient no. 7, Fig. 2, right). It may be the case that the non-compacted myocardium mimicked LVH on echocardiography, and MRI uncovered prominent LV trabeculae as the true cause of the increased LV thickness. Another cause of misdiagnosis of HCM was prior unrecognised myocardial infarction in a patient with LVH secondary to hypertension (patient no. 12, Fig. 4, bottom row). Myocardial infarction by consequent LV wall thinning causes an asymmetric pattern of hypertrophy (a thick LV wall in the area without infarction and a thin LV wall in the area of ischaemic scarring). Finally, storage disease may mimic HCM, which was the case in one patient with an established diagnosis of HCM (patient no. 8, Fig. 3, left). Further testing ultimately revealed Danon syndrome. Previous studies showed that with the use of genetic, biochemical and histopathological testing, storage diseases can be unmasked in up to 0.5–1% of patients presumed to have HCM [23–26]. In our population, we did not perform routine genetic and/or biochemical screening for HCM phenocopies. Using MRI, in our population, we identified storage/infiltrative disease in 3 out of 935 patients (0.3%) not suspected of having such a disease based on pre-MRI evaluation (including echocardiography and clinical assessment). Additionally, among patients in whom MRI was prescribed in order to differentiate between HCM and amyloidosis/storage disease, the diagnosis was confirmed in 20% of patients (14 out of 71), including 12 patients with cardiac amyloidosis and 2 patients with Fabry disease.

Alternative (to HCM) diagnoses causing LVH are made predominantly based on medical history (e.g. history of hypertension in case of hypertensive heart disease or intensive sport training in case of athlete’s heart), clinical evaluation (e.g. signs and symptoms of a systemic disease causing LVH such as Fabry disease or amyloidosis) and typical appearance in imaging tests [1, 2, 5]. However, a significant overlap exists leading to the presence of grey zones between those diagnoses especially when the disease is not advanced. In several clinical scenarios, cardiac MRI may serve as an arbitrator pointing towards a specific final diagnosis. Since asymmetric septal hypertrophy may result from several causes [5] and HCM may exhibit symmetric or diffuse LVH type [4, 27], the final diagnosis cannot be made solely on LVH pattern. Cardiac MRI employs additional features to distinguish between conditions causing LVH. Even subtle markers of cardiomyopathy such as myocardial crypts and mitral valve apparatus (leaflets, chordae, papillary muscles) abnormalities can contribute to final diagnosis [5]. We showed that when cardiac MRI with all its advantages are employed for differential diagnosis of LVH, the highest percentage of equivocal diagnosis was observed in patients with uncontrolled hypertension suspected of having HCM. This fact underscores the difficulties in clearly differentiating HCM and hypertensive heart disease. Although several recommendations have been published on how to distinguish between HCM and LVH due to hypertension [5, 7–9], incorporating these guidelines in the differential diagnosis of LVH may still result in a high percentage of patients in whom it is not possible to make an unequivocal diagnosis. Further research must clarify whether new emerging imaging techniques such as MRI-based feature tracking strain analysis or blood-oxygen-level-dependent T2* imaging would be helpful in differentiating causes of LVH [28, 29]. Additionally, advanced analysis of diastolic dysfunction by combined assessment of atrial and ventricular function in cardiac MRI should be evaluated as a potential tool for differentiating HCM and hypertensive heart disease [30].

Finally, in a small group of patients in our study, MRI aided in differentiating HCM from a cardiac tumour. Due to its ability to be used for tissue characterisation, MRI is ideally suited for this indication and can make a diagnosis with a high level of agreement with histopathological results [11]. Additionally, unrestricted MRI imaging in any desired plane, which is free from acoustic window limitations inherent for echocardiography, enables the exclusion of neoplasms in patients in whom masses observed on echocardiography turn out to be benign tumour mimics on MRI. Moreover, the situation when no cardiac mass is observed on MRI in a patient referred due to a suspected cardiac tumour is not unusual and was present in one patient in our cohort in whom MRI revealed neither LVH nor any cardiac mass. Differentiating among these situations (no pathology vs mass-like HCM phenotype vs cardiac tumour) is of particular importance for patient treatment, clinical and imaging surveillance and prognosis.

According to European guidelines and recommendations on the diagnosis and management of HCM, if local resources permit, cardiac MRI should be performed at least once in each patient with HCM [1, 5]. Our study gives evidence that every effort should be made to follow these guidelines. Several years after publishing these recommendations, a dedicated cardiac MRI unit should be regarded as an indispensable imaging facility that is complementary to echocardiography in a centre specialising in the care of HCM patients. This finding applies not only for patients with poor acoustic windows and poor ultrasound image quality but also in every patient with HCM and echocardiography-based diagnosis or suspicion of HCM. This approach concerning performing cardiac MRI in all HCM patients is justified and of important clinical impact. In a small but important group of patients with HCM established on the basis of echocardiography, MRI leads to a diagnosis different than cardiomyopathy. Additionally, when echocardiography is inconclusive, MRI is able to provide definitive diagnosis of HCM in almost 50% of the cases, in further ca. 20% of the patients to refute overt LVH and in an additional 5.3% to make a diagnosis that was different from HCM. In general, cardiac MRI should be an integral component of evaluation of patients with HCM established on the basis of echocardiogram or an ultrasound-based suspicion of cardiomyopathy, in the latter leading to clarification of diagnosis (confirming or refuting HCM) in up to three fourths of patients.

### Limitations

We did not aim to compare the use of genetic testing as a reference standard for the diagnosis of HCM. However, the main purpose of our study was not to assess the ability of cardiac MRI in the identification of genotype-positive patients but to diagnose overt disease (phenotype positive) and frank hypertrophy according to the definition of HCM. A normal MRI scan does not exclude cardiomyopathy, but neither does a negative genetic test. In a recent report on young athletes with T-wave inversion, cardiomyopathy was diagnosed mainly on the basis of MRI findings, and the diagnostic yield from genetic testing was low despite a wide panel of genes studied [31].

In contrast to the MRI examinations, echocardiograms have not been peer reviewed. Thus, we can only hypothesise that left or right ventricular trabeculations may have caused echocardiography to overestimate left ventricular wall thickness/asymmetry.

Finally, selection biases should be mentioned. Only patients without contraindications for MRI study were recruited. Additionally, HCM diagnoses or suspicions were based in all patients on echocardiography leaving those with HCM diagnosis made on the basis of cardiac computed tomography or a suspicion made basing solely on abnormal ECG findings.

## Conclusions

In a small but important group of patients with HCM, cardiac MRI can diagnose previously unknown conditions mimicking HCM and/or refute suspected cardiomyopathy based on echocardiography. The diagnostic yield of MRI in patients suspected of having HCM based on an ultrasound study is 44.7%.

## Abbreviations

HCM: Hypertrophic cardiomyopathy
LGE: Late gadolinium enhancement
LV: Left ventricular
LVEDV: Left ventricular end-diastolic volume
LVEF: Left ventricular ejection fraction
LVESV: Left ventricular end-systolic volume
LVH: Left ventricular hypertrophy
LVM: Left ventricular mass
RV: Right ventricular
SCD: Sudden cardiac death
